# Cryptanalysis and Security Improvements of ‘Two-Factor User Authentication in Wireless Sensor Networks’

**DOI:** 10.3390/s100302450

**Published:** 2010-03-23

**Authors:** Muhammad Khurram Khan, Khaled Alghathbar

**Affiliations:** 1 Center of Excellence in Information Assurance (CoEIA), King Saud University, Saudi Arabia; 2 Information Systems Department, College of Computer and Information Sciences, King Saud University, Saudi Arabia; E-Mail: kalghathbar@ksu.edu.sa

**Keywords:** authentication, wireless sensor network, security, smart card, cryptanalysis

## Abstract

User authentication in wireless sensor networks (WSN) is a critical security issue due to their unattended and hostile deployment in the field. Since sensor nodes are equipped with limited computing power, storage, and communication modules; authenticating remote users in such resource-constrained environments is a paramount security concern. Recently, M.L. Das proposed a two-factor user authentication scheme in WSNs and claimed that his scheme is secure against different kinds of attack. However, in this paper, we show that the M.L. Das-scheme has some critical security pitfalls and cannot be recommended for real applications. We point out that in his scheme: users cannot change/update their passwords, it does not provide mutual authentication between gateway node and sensor node, and is vulnerable to gateway node bypassing attack and privileged-insider attack. To overcome the inherent security weaknesses of the M.L. Das-scheme, we propose improvements and security patches that attempt to fix the susceptibilities of his scheme. The proposed security improvements can be incorporated in the M.L. Das-scheme for achieving a more secure and robust two-factor user authentication in WSNs.

## 1. Introduction

With the recent advances in communication technologies, wireless sensor networks (WSN) have emerged as a very active research avenue. WSNs have many common features with wireless ad hoc networks, and in several cases they are considered as a special case of them [1]. A WSN usually consists of a large number of autonomous sensor nodes, which are generally deployed in unattended environments. Each sensor node has some level of computing power, limited storage, and a small communication module to communicate with the outside world over an ad hoc wireless network [2]. WSNs are widely used, including in areas such as military, battlefield, homeland security, healthcare, environment monitoring, agriculture and cropping, manufacturing, *etc*.

Since the sensor network may operate in a hostile environment such as a military battlefield, security is critical. Robust techniques are needed to provide low-latency, survivable, and secure networks during the deployment of WSN. In addition, the network should be protected against intrusions and spoofing attacks [3]. Access control is an indispensable cryptographic primitive upon which other security primitives are built. A WSN should be smart enough to distinguish legitimate users from illegitimate users, resulting in the problem of user authentication [3]. If a WSN is deployed for a highly secure application, then the data collected within the sensor work is valuable and should only be given access to the registered or legitimate users. Benenson *et al.* first sketched the security issues of user authentication in WSN and introduced the notion of *n-authentication* [4]. Later on, Watro *et al.* proposed a TinyPK authentication protocol with public key cryptography that uses RSA and Diffie-Hellman algorithms [5], however, this protocol suffers from masquerade sensor node attack, in which an adversary can spoof the user.

In 2006, Wong *et al.* [6] proposed a light-weight dynamic user authentication scheme in WSN environment. They justified their scheme through security and cost analysis and discussed the implementation issues with the recommendations of using the security features of IEEE 802.15.4 MAC sublayer. Later, Tseng *et al.* [7] identified some security weaknesses in the scheme of Wong *et al.,* which prevent it from being implemented in real-life environments. They showed that Wong *et al.*’s scheme is not protected from replay and forgery attacks, passwords can easily be revealed by any of the sensor nodes, and users cannot freely change their passwords. To overcome these discrepancies, Tseng *et al.* proposed an enhanced scheme and claimed that their scheme not only retains the advantages of Wong *et al.*’s scheme, but provides: resistance to replay and forgery attacks, reduction of password leakage risk, and capability of changeable password with better efficiency [7]. Lately, T.H. Lee [8] also analyzed Wong *et al.*’s scheme and proposed two simple dynamic user authentication protocols that are variations of Wong *et al.*’s scheme. In his first protocol, T.H. Lee simplified the authentication process by reducing the computational load of sensor nodes while preserving the same security level of Wong *et al.*’s scheme. On the other hand, in his second protocol, T.H. Lee proposed a scheme in which an intruder cannot impersonate the gateway node to grant access to illegitimate users.

L.C. Ko [9] proved that while Tseng *et al.*’s scheme achieves several security measures above Wong *et al.*’s scheme, it is still insecure under a reasonable attack model [9]. L.C. Ko discussed that Tseng *et al.*’s scheme does not achieve mutual authentication between the Gateway node *(GW)* and the Sensor node *(SN)*, and between the User *(U)* and the SN. Furthermore, L.C. Ko identified that an adversary can forge the communication message which is sent from sensor node to the gateway node. Consequently, L.C. Ko proposed a modified scheme which attempts to overcome the aforementioned security pitfalls of Tseng *et al.*’s protocol and proved that his scheme has better security features than Tseng *et al.*’s scheme. [7]

Binod *et al.* [10] cryptanalyzed the authentication schemes of Wong *et al.* and Tseng *et al.* and proposed their improved scheme. Binod *et al.* showed that their scheme is more robust than previously published schemes and can withstand replay attack, forgery attack, man-in-the-middle attack and provides mutual authentication between login node and gateway node.

Recently, M.L. Das [11] proposed a two-factor user authentication scheme in WSNs. M.L. Das also identified that Wong *et al.*’s protocol is vulnerable to many logged-in users with the same login-id threat, that is, who has a valid user’s password can easily login to the sensor network [11]. He also identified that Wong *et al.*’s protocol is susceptible to stolen-verifier attack, because the GW-node and login-node maintain the lookup table of all the registered users’ credentials. Consequently, M.L. Das proposed his protocol to overcome the security flaws of Wong *et al.*’s scheme. His protocol uses the two factor authentication concept based on password and smart card and resists many logged-in users with the same login identity, stolen-verifier, guessing, replay, and impersonation attacks.

More recently, Nyang and Lee pointed out that the protocol of M.L. Das is vulnerable to offline password guessing attack, sensor node compromising attack, and does not protect query response messages by establishing a unique secure channel from sensor node to a user, which is an important way of serving a registered user in a secure and legitimate way [17]. Consequently, Nyang and Lee proposed their improved two-factor authentication protocol for WSNs, which attempts to overcome their identified discrepancies in the M.L. Das scheme.

However, in this paper, we identify that the M.L. Das-scheme is still not secure and vulnerable to several critical security attacks. In addition to the problems identified by Nyang and Lee, we show that the M.L. Das-scheme is defenseless against GW-node by-passing attack, does not provide mutual authentication between GW-node and sensor nodes, has the security threat of insider attack, and does not have provision for changing or updating passwords of registered users. To fix the aforementioned weaknesses of the M.L. Das-scheme, we propose security improvements in our paper. Our enhanced security patch contains secure features of changing or updating passwords of users, provides protection against insider attack, overcomes the GW-node bypassing attack, and provides mutual authentication between GW-node and sensor node. The proposed security improvements can easily be incorporated into the M.L. Das-scheme to take the benefit of more secure and robust two-factor user authentication in WSNs.

The rest of the paper is organized as follows; Section 2 briefly reviews the M.L. Das-scheme, Section 3 elaborates on the weaknesses and security pitfalls of his scheme, Section 4 presents our proposed security patch, improvements and analysis over the M.L. Das-scheme, Section 5 reveals the performance analysis of the presented scheme, and finally, Section 6 concludes this paper.

## 2. Review of the M.L. Das-Scheme

In this section, we briefly review user the authentication scheme of M.L. Das, which is divided into two phases, namely the registration phase and the authentication phase.

### 2.1. Registration Phase

When a user *U_i_* wants to perform registration with the WSN, he submits his *ID_i_* and *pw_i_* to the Gateway node (GW-node) in a secure manner. Upon receiving the registration request, the GW-node computes *N_i_* = *h*(*ID_i_*||*pw_i_*) ⊕ *h*(*K*), where *K* is a symmetric key that is secure to the GW-node, and ‘||’ is a bit-wise concatenation operator. Now, the GW-node personalizes the smart card with the parameters *h*(.), *ID_i_*, *N_i_*, *h*(*pw_i_*) and *x_a_*, where *h*(.) is a one-way secure hash function and *x_a_* is a secret value generated securely by the GW-node and stored in some designated sensor nodes before deploying the WSN. At the end of this phase, *U_i_* gets his personalized smart card in a secure manner.

### 2.2. Authentication Phase

The authentication phase is invoked when *U_i_* wants to login into WSN or access data from the network. This phase is further sub-divided into two phases, namely login and verification phases.

1. *Login Phase*
  In the login phase, *U_i_* inserts his smart card into terminal and inputs *ID_i_* and *pw_i_*. The smart card validates the *ID_i_* and *pw_i_* with the stored values. If *U_i_* is successfully authenticated, the smart card performs the following steps:
    Step- L1: Computes *DID_i_* = *h*(*ID_i_*||*pw_i_*) ⊕ *h*(*x_a_*||*T*), where *T* is the current timestamp of *U_i_* system
    Step- L2: Computes *C_i_* = *h*(*N_i_*||*x_a_*||*T*), then send < *DID_i_*, *C_i_*, *T* > to the GW-node
2. *Verification Phase*
  Upon receiving the login request < *DID_i_*, *C_i_*, *T* > at time T*, the GW-node authenticates *U_i_* by the following steps:
    Step-V1: Checks if (T* − *T*) ≤ Δ*T* then GW-node proceeds to the next step, otherwise verification step is terminated. Here Δ*T* shows the expected time interval for the transmission delay
    Step-V2: Computes *h*(*ID_i_*||*pw_i_*)* = *DID_i_* ⊕ *h*(*x_a_*||*T*) and $C_{i}^{*}=h\left(\left(h{\left({\mathrm{ID}}_{i}||{\mathrm{pw}}_{i}\right)}^{*}\;⊕\;\;h(K)\right)\left\|x_{a}\right\|T\right)$
    Step-V3: if $C_{i}^{*}=C_{i}$ then GW-node accepts the login request; otherwise login request is rejected.
    Step-V4: GW-node now sends a message < *DID_i_*, *A_i_*, *T′* > to some nearest sensor *S_n_* over a public channel to respond the query data what *U_i_* is looking for, where the value of *A_i_* is *A_i_* *= h*(*DID_i_*||*S_n_*||*x_a_*||*T*′), where *T*′ is the current timestamp of the GW-node. Here, the value of *A_i_* is used to ensure *S_n_* that the message originally comes from the real GW-node.
    Step-V5: After receiving the message < *DID_i_*, *A_i_*, *T′* >, the *S_n_* validates the timestamp. If the timestamp is within valid interval, then *S_n_* computes *h*(*DID_i_*||*S_n_*||*x_a_*||*T*′) and checks whether it is equal to *A_i_*. If this step is passed, then *S_n_* responds to the *U_i_*’s query.

## 3. Cryptanalysis and Security Pitfalls of the M.L. Das-Scheme

### 3.1. GW-Node Bypassing Attack

In the M.L. Das-scheme, after performing the verification phase and accepting the login request of *U_i_*, the GW-node sends an intimation message < *DID_i_*, *A_i_*,*T′* > to some nearest sensor node *S_n_* to inform about the successful login of *U_i_*, and requests *S_n_* to respond the query/data of *U_i_*. Here, *A_i_* is computed by *A_i_* *= h*(*DID_i_*||*S_n_*||*x_a_*||*T*′), where *x_a_* is a secret parameter which is known to GW-node, sensor node and stored in the smart card of *U_i_*. *T*′is the timestamp of GW-Node and *DID_i_* is the dynamic ID of user, which is calculated by *DID_i_* = *h*(*ID_i_*||*pw_i_*) ⊕ *h*(*x_a_*||*T*). In the M.L. Das-scheme, the value of *x_a_* is used to ensure *S_n_* that *A_i_* message is coming from the legitimate GW-node. Here, we assume that if the value of *x_a_* is extracted from smart card of *U_i_* by some means [12,13], then *U_i_* himself or any adversary can login the *S_n_* without going through the verification of GW-node, so Das *et al.*’s scheme is vulnerable to ‘GW-node by-passing attack’. In the following, we show how this attack works on the M.L Das-scheme:

- Suppose an adversary or *U_i_* himself computes a fake dynamic identity *DID_a_* by using the extracted *x_a_* from smart card *DID_f_* = *h*(*ID_f_* ||*pw_f_*) ⊕ *h*(*x_a_*||*T_f_*), where *ID_f_* is a fake ID of adversary, *pw_f_* is a randomly chosen fake password, and *T_f_* is the current timestamp of adversary’s machine.
- Adversary computes *A_f_* *= h*(*DID_f_*||*S_n_*||*x_a_*||*T_f_*), where *S_n_* is the nearest sensor node for querying the data.
- Now, adversary sends the message < *DID_f_*, *A_f_*, *T_f_* > to *S_n_* over insecure communication channel.
- After receiving the message, *S_n_* first validates *T_f_*. If (T* − *T_f_*) ≤ Δ*T*, then *S_n_* proceeds to next step, otherwise terminates the operation. Here, Δ*T* shows the expected time interval for the transmission delay.
- *S_n_* now computes *A′_f_* *= h*(*DID_f_*||*S_n_*||*x_a_*||*T_f_*) and checks whether the value of ${A^{\prime }}_{f}\overset{?}{=}A_{f}$ or not. If it holds, *S_n_* responds to the adversary’s query, and *U_a_*, who is an adversary and not a legitimate user of the sensor network system, enjoys the resources as an authorized user without being a member of the system.

### 3.2. No Mutual Authentication between GW and Sensor Nodes

In the M.L. Das-scheme, after accepting the login request of *U_i_*, the GW-node sends a message < *DID_i_*, *A_i_*, *T′* > to some nearest sensor node *S_n_*. Here the value of *A_i_* is computed by *A_i_* *= h*(*DID_i_*||*S_n_*||*x_a_*||*T*′), where *T*′ is the current timestamp of GW-node. This message informs the sensor node to respond the query/data, which *U_i_* is requesting from the sensor network. In this message, the value of *A_i_* is used to ensure the sensor node that it is come from the real GW-node. However, sensor node verifies the authenticity of GW-node but there is no authenticity that the sensor node is fake or real. Thus, the M.L. Das-scheme only provides unilateral authentication between the GW-node and sensor node, and there is not mutual authentication between the two nodes, which is an indispensable property of authentication protocol designing [14].

### 3.3. Privileged-Insider Attack

In a real environment, it is a common practice that many users use same passwords to access different applications or servers for their convenience of remembering long passwords and ease-of-use whenever required. However, if the system manager or a privileged-insider of the GW-node knows the passwords of *U_i_*, he may try to impersonate *U_i_* by accessing other servers where *U_i_* could be a registered user. In the M.L. Das-scheme, *U_i_* performs registration with GW-node by presenting his password in plain format *i.e., pw_i_*. Thus, his scheme has pitfalls in terms of insider’s attack of GW-node by a privileged user who has come to know the password of *U_i_* and can misuse the system in future [15].

### 3.4. No Provision for Changing/Updating Passwords

In the M.L. Das-scheme, there is no provision for *U_i_* to change or update his password whenever required. It is widely recommended security policy for highly secure applications that user’s should update or change their passwords frequently, while there is no such option in the M.L. Das-scheme.

## 4. Proposed Security Improvements and Analysis

In this section, we propose security improvements over the scheme of M.L. Das and perform analysis of our security patches as follows:

### 4.1. Introducing Password Change Phase

In this subsection, we introduce the password-change/update phase in the M.L. Das-scheme. In the password-change phase, when a user wants to change his password *pw_i_* to a new password $pw_{i}^{*}$, he inserts his smart card into the terminal and enters his ID and password. Smart card validates his *ID_i_* and *pw_i_* with the stored values and if the entered *ID_i_* and *pw_i_* are correct, then the smart performs the following operations without interacting with GW-node:

- Computes $N_{i}^{*}=N_{i}⊕h\left({\mathrm{ID}}_{i}||{\mathrm{pw}}_{i}\right)⊕h\left({\mathrm{ID}}_{i}||{\mathrm{pw}}_{i}^{*}\right)$, where the value of *N_i_* is already stored on smart card i.e. *N_i_* = *h*(*ID_i_*||*pw_i_*) ⊕ *h*(*K*)
- Smart card replaces the old value of *N_i_* with the new values $N_{i}^{*}$ and $h\left({\mathrm{pw}}_{i}^{*}\right)$. Now, the new password is successfully changed and this phase is terminated.

### 4.2. Protection against Insider Attack

As we have mentioned in subsection 3.3, the M.L. Das-scheme has vulnerability of privileged-insider attack due to the reason of presenting his plain text password *pw_i_* to the GW-node. This problem can simply be overcome if *U_i_* only submits *h*(*pw_i_*) to the GW-node, which is the hashed value of plain text password. Thus in the registration phase, the GW-node would compute *N_i_* = *h*(*ID_i_*||*h*(*pw_i_*)) ⊕ *h*(*K*), instead of just *N_i_* = *h*(*ID_i_*||*pw_i_*) ⊕ *h*(*K*), and the person except *U_i_* will never know his secret password, which can protect from the possibility of privileged-insider attack [16].

### 4.3. Overcoming GW-node Bypassing Attack and Providing Mutual Authentication

It was identified in subsection 3.1 that there is the possibility of GW-node bypassing attack in M.L. Das-scheme and an adversary without passing the login from the GW-node can access the resources of the sensor network. The reason for the possibility of GW-node bypassing attack is due to sharing of secret parameter *x_a_* with the sensor node *S_n_* and user *U_i_*. If the value of *x_a_* is compromised, then the whole sensor network will become vulnerable to the GW-node bypassing attack.

Thus, we propose not to share the same secret parameters with *S_n_* and *U_i_*, and that every entity has its own secret parameter or key. Here, we suggest that the GW-node should only share *x_a_* with *U_i_* and there should be another secret parameter *x_s_*, which should only be known to the GW-node and sensor nodes, and can be stored in sensor nodes before their deployment in the field. These sensor nodes are responsible to respond users for their queries.

To overcome this security flaw, the Step-V4 and Step-V5 in the verification phase of the M.L. Das-scheme can be amended by the following steps:

- After accepting the login request of *U_i_*, the GW-node sends message < *DID_i_*, *A_i_*, *T′* >, to some nearest sensor node *S_n_* to respond the query/data of *U_i_*, where *A_i_* is computed by *A_i_ = h*(*DID_i_*||*S_n_*||*x_s_*||*T*′). Here *x_s_* is the secret parameter, which is securely stored in sensor node *S_n_* and shared only with the GW-node, and *T*′ is the current timestamp of GW-node’s system.
- Upon receiving the message < *DID_i_*, *A_i_*, *T′* >, the designated sensor node validates the timestamp. If (*T″* − *T′*) ≤ Δ*T*, then *S_n_* proceeds to next step, otherwise terminates the further operation. Here, Δ*T* shows the expected time interval for the transmission delay and *T″* is the current timestamp of sensor node *S_n_*.
- *S_n_* now computes $A_{i}^{*}=h\left({\mathrm{DID}}_{i}\|S_{n}\left\|x_{s}\right\|T^{\prime }\right)$ and checks whether $A_{i}^{*}\overset{?}{=}A_{i}$ or not. If it holds, then *S_n_* responds to *U_i_*’s query, otherwise terminates the operation.
- To provide mutual authentication between GW-node and sensor node, *S_n_* now computes *B_i_ = h*(*S_n_*||*x_s_*||*T″′*). Here T*″′* is the current timestamp of sensor node’s system and sends back mutual authentication message < *B_i_*, *T″′* > to the GW-node.
- After receiving the mutual authentication message < *B_i_*, *T″′* >, the GW-node first checks the validity of time-stamp. If (*T″″* − *T″′*) ≤ Δ*T*, then GW node performs the further operations, otherwise the mutual authentication phase is terminated. Here, Δ*T* shows the expected time interval for the transmission delay and *T″″* is the current timestamp of GW-node.
- GW-node now computes $B_{i}^{*}=h\left(S_{n}\left\|x_{s}\right\|{{T^{\prime }}^{\prime }}^{\prime }\right)$ and checks whether $B_{i}^{*}\overset{?}{=}B_{i}$ or not. If it is true, then GW-node establishes trust on sensor node, otherwise, GW-node intimates *U_i_* about the possibility of malicious sensor node in the network and sends a process-termination message.
- After successful authentication, *U_i_* enjoys the resources provided by the sensor network.

Although, in the proposed security patch, the introduction of one more secret parameter *x_s_* creates storage overhead on the GW-node, but its benefits are two-fold and cannot be overlooked. The first benefit, as defined previously, is to overcome the GW-node bypassing attack, while the second benefit is the ease of secret parameter (key) updating incase of compromise of *x_s_* by an adversary. In the M.L. Das- scheme, if *x_a_* is compromised and GW-node has to revoke *x_a_* with a new secret parameter *x′_a_*, then the cost of revoking *x′_a_* is very high because it needs to be updated on all *U_i_*’s smart cards as well as all the sensor nodes in the field. While on the other hand, in our proposed security improvement/patch, the cost of revoking secret parameters either *x_a_* or *x_s_* can be halved due to assigning different values *x_a_* and *x_s_* to *U_i_* and *S_n_*, respectively.

## 5. Performance Analysis of Proposed Scheme

In this section, we summarize security features and performance analysis of our proposed scheme and compare its security and robustness with the schemes of M.L. Das [11], and Nyang and Lee [17]. Table 1 demonstrates that our scheme is more secure and robust than the schemes of [11] and [17], and achieves more security features, which were not considered in the aforementioned schemes and are essentially required to implement a practical and universal two-factor user authentication protocol in WSNs.

Furthermore, it can be seen from Table 1 that our scheme needs only 13 hashing operations, in contrast to the protocols of M.L. Das and Nyang-Lee, which require 10 and 17 hash computations, respectively. Our scheme provides protection against insider attack, gateway node bypassing attack, password change/update option, and achieves mutual authentication between gateway and sensor nodes, which require few more hashing operations than [11] to enhance the security of overall authentication system. Hence, the computational overhead of the proposed scheme are not too high, but the scheme contains several enhanced security features, which are indispensable for implementing a reliable and trustworthy remote user authentication scheme in the WSN environment.

## 6. Conclusions

In this paper, we have shown that a recently proposed two-factor user authentication scheme in WSN environment is insecure against different kinds of attack and should not be implemented in real-applications. We have demonstrated that in the M.L. Das-scheme, there is no provision for users to change or update their passwords, the GW-node bypassing attack is possible, it does not provide mutual authentication between GW-node and sensor node, and it is susceptible to privileged-insider attack. To remedy the aforementioned flaws, we have proposed security patches and improvements, which overcome the weak features of the M.L. Das-scheme. The presented security improvements can easily be incorporated in the M.L. Das-scheme for a more secure and robust two-factor user authentication in WSNs.

## Figures and Tables

**Table 1.** Performance analysis and comparison of the proposed scheme.

**Security Features and Performance**	**Proposed scheme**	**M.L. Das [11]**	**Nyang-Lee [17]**
Securely change/update password	Yes	No	No
Protection against insider’s attack	Yes	No	No
Protection against Gateway node bypassing attack	Yes	No	No
Mutual authentication between GW and sensor nodes	Yes	No	Yes
Computational operations in registration phase	3H	2H	2H
Computational operations in login phase	3H	3H	3H
Computational operations in verification phase	7H	5H	12H

H: *The computational cost of one hash operation*
